# Promoting intestinal antimicrobial defense and microbiome symbiosis contributes to IL-22-mediated protection against alcoholic hepatitis in mice

**DOI:** 10.3389/fimmu.2023.1289356

**Published:** 2023-10-16

**Authors:** Ruichao Yue, Xiaoyuan Wei, Liuyi Hao, Haibo Dong, Wei Guo, Xinguo Sun, Jiangchao Zhao, Zhanxiang Zhou, Wei Zhong

**Affiliations:** ^1^ Center for Translational Biomedical Research, University of North Carolina at Greensboro, Kannapolis, NC, United States; ^2^ Department of Animal Science, Division of Agriculture, University of Arkansas, Fayetteville, AR, United States; ^3^ Department of Nutrition, University of North Carolina at Greensboro, Greensboro, NC, United States

**Keywords:** Alcohol-associated liver disease, IL-22, STAT3, antimicrobial peptide, microbiota, PAMP translocation

## Abstract

**Background:**

The hepatoprotective effect of interleukin 22 (IL-22) has been reported in several models of liver injuries, including alcohol-associated liver disease (ALD). However, the intestinal role of IL-22 in alcoholic hepatitis remains to be elucidated.

**Methods:**

Intestinal IL-22 levels were measured in mice fed with alcohol for 8 weeks. IL-22 was then administered to alcohol-fed mice to test its protective effects on alleviating alcoholic hepatitis, focusing on intestinal protection. Acute IL-22 treatment was conducted in mice to further explore the link between IL-22 and the induction of antimicrobial peptide (AMP). Intestinal epithelial cell-specific knockout of signal transducer and activator of transcription 3 (STAT3) mice were generated and used for organoid study to explore its role in IL-22-mediated AMP expression and gut barrier integrity.

**Results:**

After alcohol feeding for 8 weeks, the intestinal levels of IL-22 were significantly reduced in mice. IL-22 treatment to alcohol-fed mice mitigated liver injury as indicated by normalized serum transaminase levels, improved liver histology, reduced lipid accumulation, and attenuated inflammation. In the intestine, alcohol-reduced Reg3γ and α-defensins levels were reversed by IL-22 treatment. IL-22 also improved gut barrier integrity and decreased endotoxemia in alcohol-fed mice. While alcohol feeding significantly reduced *Akkermansia*, IL-22 administration dramatically expanded this commensal bacterium in mice. Regardless of alcohol, acute IL-22 treatment induced a fast and robust induction of intestinal AMPs and STAT3 activation. By using *in vitro* cultured intestinal organoids isolated from WT mice and mice deficient in intestinal epithelial-STAT3, we further demonstrated that STAT3 is required for IL-22-mediated AMP expression. In addition, IL-22 also regulates intestinal epithelium differentiation as indicated by direct regulation of sodium-hydrogen exchanger 3 via STAT3.

**Conclusion:**

Our study suggests that IL-22 not only targets the liver but also benefits the intestine in many aspects. The intestinal effects of IL-22 include regulating AMP expression, microbiota, and gut barrier function that is pivotal in ameliorating alcohol induced translocation of gut-derived bacterial pathogens and liver inflammation.

## Introduction

Alcohol is a major causal factor for the burden of disease worldwide. Heavy and prolonged alcohol drinking damages multiple organs, especially the liver (1, 2). Alcohol-associated liver disease (ALD) covers a spectrum of liver disorders involves reversible steatosis, hepatitis, fibrosis, to irreversible cirrhosis and eventually liver failure (1, 2). Excessive alcohol drinking leads to the translocation of gut microbiota-derived pathogen associated molecular patterns (PAMPs), such as lipopolysaccharides (LPS), to the bloodstream, which reach the liver to elicit an inflammatory cascade and the development of alcoholic hepatitis (AH) (3, 4). Early studies have observed changes in the compositions of gut microbiota (dysbiosis) in both patients with AH and animal models of AH (5–7). Furthermore, transplantation of gut microbiota from AH patients to mice directly induced severe liver damage strongly suggest a causal link between mediators derived from alcohol-perturbed gut microbiota and disease progression (8). Therefore, investigation regarding the mechanisms of alcohol-induced gut dysbiosis and the underlying molecular targets are necessitated for prevention and therapy purposes.

Intestinal antimicrobial peptides (AMPs) are a variety of small peptides produced by intestinal epithelial cells (IECs) and specialized AMP-secreting cells named Paneth cells. They act as the first line of defense to protect the host against infections caused by bacteria, viruses, fungi, or parasites (9, 10). Besides having a direct antimicrobial activity, some AMPs exhibit the ability to regulate host immune response and thereby indirectly eliminate infection (11, 12). The critical role of AMPs in innate immunity is increasingly recognized in AH. Regenerating islet-derived protein 3β (Reg3β) and Reg3γ were found to be reduced in the intestines of mice after alcohol consumption, and overexpression of Reg3γ restricted alcohol-induced PAMP translocation and alleviated liver damage (13). Recently, our group reported that α-defensins, another type of AMPs, that are exclusively produced by Paneth cells are significantly decreased in a mouse model of AH (6). We further revealed the therapeutic potential of AMPs in treating AH as synthetic human α-defensin 5 (HD5) treatment corrected alcohol-perturbed gut microbiota, alleviated alcohol-induced gut barrier disruption and endotoxemia, and ultimately improves AH in mice. Despite the emerging attention to AMPs in orchestrating gut microbiota symbiosis and combatting AH, it remains unclear how alcohol alters the expression and secretion of AMPs.

Interleukin-22 (IL-22) is a cytokine produced by type 3 innate lymphoid cells (ILC3) and T cells in the intestine (14, 15). It is critically involved in proliferation, regeneration, and cellular defense (16). One of the working mechanisms for IL-22 in maintaining a healthy gut is via the induction of several AMPs, including Reg3 family, lipocalin-2, S100 proteins, and β-defensins (16, 17), which are all primarily produced by IECs. Importantly, intestinal IL-22 levels were reduced in the chronic-binge alcohol feeding (NIAAA) mouse model (18). In that study, the authors constructed a *Lactobacillus reuteri* (*L. reuteri*) strain overexpressing IL-22 and found that IL-22 overexpression induced Reg3γ and reduced alcohol-induced liver damage. The significance of IL-22 at the gut-liver-axis in AH was also suggested by another study, which demonstrated that toll-like receptor 7 (TLR7) agonist-mediated protection was abolished by IL-22 deficiency in a mouse model of AH (19). However, the specific AMP targets of IL-22 still remain largely unknown, especially whether IL-22 instructs the antimicrobial function of Paneth cells and/or directly affects Paneth cell AMP production. Recently, Paneth cells were found to express IL-22 receptor, IL-22RA1, and mice with Paneth cell-specific Il22ra1 deficiency were susceptible to bacterial infection (20). As a result, IL-22 can modulate gut microbiota by indirectly shaping their compositions via AMP production (21) or by directly promoting the growth of commensal communities (22, 23). It is, however, still unclear whether and how IL-22 orchestrates gut microbiota in mice exposed to alcohol.

IL-22 interacts with the IL-22 receptor complex named IL-22RA1 and IL-10R2, to phosphorylate and activate the signal transducer and activator of transcription (STAT) pathway, including STAT1, STAT3, and STAT5 (24). Emerging evidence suggest that epithelial STAT proteins participate in host immune defense against bacterial or viral infections as well as tissue regeneration (25–27). *Citrobacter rodentium* infection is observed with IL-22 elevation, epithelial STAT3 activation, and the induction of Reg3γ (28). In our previous study, we found that alcohol consumption deactivated intestinal STAT3 in mice, which could be reversed by treatment with IFN-γ (29).

In this study, we determined the role of IL-22 in reversing the detrimental effects of alcohol on the intestine and liver. We aimed to explore the effects of IL-22 in improving alcohol-impaired intestinal AMP production, gut microbiota symbiosis, epithelial barrier, and the subsequent PAMP translocation and liver damage. A mouse model with IEC-specific knockout of STAT3 was used to investigate the role of STAT3 in IL-22-mediated AMP production *in vivo* and *in vitro*.

## Materials and methods

### Mice

Mice with a floxed STAT3 allele (Stat3^fl/fl^; Stock No. 016903) were purchased from the Jackson Laboratory (Bar Harbor, ME). Intestinal epithelial cell (IEC)-specific STAT3 (Stat3^IEC-/-^) mice were generated by breeding corresponding floxed mice with Villin997-Cre mice (the Jackson Laboratory; Stock No. 004586). Wild type (WT) C57BL/6J mice (Stock No. 000664) were also obtained from the Jackson Laboratory. All mice were kept in ventilated cages under specific pathogen-free condition. Animal experiments were approved by the Institutional Animal Care and Use Committee of North Carolina Research Campus (Protocol #21-008).

### Chronic alcohol feeding and IL-22 treatments

Twelve week-old male WT C57BL/6J mice were subjected to Lieber-DeCarli liquid diets for 8 weeks as previously described (30). Briefly, alcohol-fed mice (AF; n = 6) were given alcohol liquid diet and control mice were pair-fed (PF; n = 6) an isocaloric control lipid diet. Food grade ethanol was purchased from Sigma-Aldrich (St. Louis, MO) and added to the diet at 4.00, 4.14, 4.28, or 4.42% for every quarter (two-week interval). IL-22 intervention was introduced to AF mice through intraperitoneal injection of recombinant mouse IL-22 (Biolegend, San Diego, CA; Cat. #576208; n = 6) at 1 mg/kg every other day for the last 2 weeks of alcohol feeding.

To test the time effects of IL-22 on AMPs, male WT mice were intraperitoneally treated with either recombinant mouse IL-22 at 1 mg/kg or same volume of saline for 1, 3, or 8 h (n = 6 per group). Tissue samples were collected after isoflurane anesthesia in strict accordance with the protocols approved.

### Organoid culture and treatments

Small intestinal crypts were isolated from PF and AF mice as well as from Stat3^fl/fl^ and Stat3^IEC-/-^ mice following a method reported by Sato et al. (31) and cultured in Matrigel (Corning, Corning, NY). Morphology of organoids was examined under light microscope during culture. After 6 days of growth, organoids were incubated with 100 ng/ml IL-22 for 10 h and harvested for gene expression and protein localization analysis by relative PCR and immunofluorescence staining, respectively.

### Biochemical and histopathological analysis of liver damage

Liver damage caused by alcohol consumption were analyzed by serum transferase levels (alanine aminotransferase/ALT and aspartate aminotransferase/AST), liver histology, and hepatic lipid accumulation. Serum ALT and AST levels were quantified by Thermo Fisher Scientific Infinity Reagents (Waltham, MA), respectively. For histological examination of the liver, paraffin-embedded liver sections were stained with hematoxylin and eosin (H&E; Dako-Agilent, Santa Clara, CA). Quantification assays of triglycerides (TG) and free fatty acids (FFA) in the liver were conducted using commercial kits from Abcam (Waltham, MA; Cat. #ab65336 and ab65341) per the manufacturer’s instructions. Hepatic neutral lipid droplets were examined by boron-dipyrromethene (BODIPY) staining. Liver cryostat sections were incubated with 1 µg/ml BODIPY 493/503 (Thermo Fisher Scientific) for 20 min at room temperature followed by DAPI (4′,6-diamidino-2-phenylindole; Thermo Fisher Scientific) counterstaining after routine fixation and permeabilization.

### Lipopolysaccharide quantification

Lipopolysaccharide (LPS) levels in the blood were determined using a Limulus amoebocyte lysate (LAL) method-based assay kit from Thermo Fisher Scientific, whereas LPS in the livers of mice was quantified using EndoLISA Endotoxin Detection Kit (BioVendor, Asheville, NC; Cat. #609033) as recommended by the manufacturer’s protocol. The levels of serum endotoxin were expressed in endotoxin units (EU) per milliliter, whereas the levels of hepatic endotoxin were expressed as EU per milligram.

### Sequencing and analysis of the bacterial 16S amplicons

Mouse cecal contents DNAs were extracted using DNeasy PowerLyzer PowerSoil kit (Qiagen, Germantown, MD; Cat. #12855) and sequenced for the V4 region of the 16s rRNA genes on Illumina MiSeq platform (Illumina Inc., San Diego, CA) (6). The acquired 16S rRNA MiSeq data were analyzed by Mothur software (v.1.39.5) (32), quality-filtered, aligned against SILVA v132 database (33), clustered into operational taxonomic units (OTU), and classified against the Ribosomal Database Project (34). Alpha diversity was calculated by observed OTUs and Shannon index. Beta diversity among the samples were explored by Bray-Curtis distance matrices. Reconstruction of unobserved states (PICRUSt) was used to predict functional genes of microbiota based on taxonomy obtained from the Greengenes reference database (35). The categories with log linear discrimination analysis (LDA) scores of > 3.0 were considered as differential signatures than better discriminate between AF and AF+IL-22 groups. Then linear discrimination analysis effect size (LEfSe) was used to explore functional genes between groups. Lastly, the bacterial correlations coefficient in PF and AF samples with and without IL-22 treatment were calculated based on the relative abundance of each genus using Spearman. Cytoscape 3.9.0 was used for co-occurrence network building and analyzed network density and network centralization.

### Gene expression analysis

RNAs of ileal or liver tissue were isolated using TRIzol reagent (Thermo Fisher Scientific) and reverse transcribed using TaqMan Reverse Transcription Reagents (Thermo Fisher Scientific; Cat. #N8080234) as recommended by the manufacturer. Relative PCR was performed using SYBR green PCR master mix (Qiagen; Cat. #330523) on a QuantStudio 5 real-time RT-PCR system. Samples were normalized to the general housekeeping gene *Rn18s* and calculated as relative fold change to controls (36). Primers summarized in **Table 1** were synthesized by Integrated DNA Technologies (Coralville, CA).

**Table 1:** Primer sequences used for qPCR analysis.

Gene	Genebank Accession No.	Forward Primer (5´-3´)/ Reverse Primer (5´-3´)
Il22	NM_016971	AGCTTGAGGTGTCCAACTTC CCGGACATCTGTGTTGTTATCT
Il22ra1	NM_178257	CATCCTCTCCACTCCCAAATAC CACCTAAGGAGGTGACTTTCTG
Dgat1	NM_010046	GGCCTTACTGGTTGAGTCTATC GTTGACATCCCGGTAGGAATAA
Dgat2	NM_026384	GAAGGGCTTCTCTTCTCTTCAC CTTTCTCCCAACGCCTCATAA
Cd36	NM_007643	GGAGTGCTGGATTAGTGGTTAG GCTGTGAGCAGACGTATAGAAG
Fatp5	NM_009512	CTACGCTGGCTGCATATAGATG CCACAAAGGTCTCTGGAGGAT
Fabp1	NM_017399	CCAGAAAGGGAAGGACATCAA ACTCATTGCGGACCACTTT
Cpt1α	NM_013495	CTCCGCCTGAGCCATGAAG CACCAGTGATGATGCCATTCT
Acox1	NM_015729	CGCACATCTTGGATGGTAGT GGCTTCGAGTGAGGAAGTTATAG
Tnf	NM_013693	GAAGTTCCCAAATGGCCTCC GTGAGGGTCTGGGCCATAGA
Ccl2	NM_011333	CAGGTCCCTGTCATGCTTCT TCTGGACCCATTCCTTCTTG
Sele	NM_011345	CACAGGAACACCCTGACTATG CCAAAGGAGCAGGAGGAATTA
Lbp	NM_008489	CAGATCCGCAAGGACTTCTTAT CCACTGAGACCCATCTTTCTTC
Cd14	NM_009841	CTGGCACAGAATGCCCTAAT TTCCTCCTAACAGCCCTACTC
Reg3b	NM_011036	AATGGAGGTGGATGGGAATG CCACAGAAAGCACGGTCTAA
Reg3g	NM_011260	TTCTCAGGTGCAAGGTGAAG GGCATAGCAATAGGAGCCATAG
Defa4	NM_010039	CCAGGGGAAGATGACCAGGCTG TGCAGCGACGATTTCTACAAAGGC
Defa5	NM_007851	CAGGCTGATCCTATCCACAAA CTTGGCCTCCAAAGGAGATAG
Defa24	NM_001024225	CAGGCTGTGTCTGTCTCTTT GCAGCCTCTTGCTCTACAATA
Mmp7	NM_010810	GAGTGCCAGATGTTGCAGAATA ATCCACTACGATCCGAGGTAAG
Nhe3	NM_001081060	CTGGCTTCGTCTTTGTCATTTC GTTGGCCTTCACGTACTTCT
Rn18s	NR_046237	ACGGACCAGAGCGAAAGCAT TGTCAATCCTGTCCGTGTCC

### Western blot

Mouse ileal protein lysates were extracted by T-PER Protein Extraction Reagent (Fisher Scientific) containing 1% protease inhibitors (Sigma-Aldrich). Aliquots containing 30 µg of total proteins were separated by sodium dodecyl sulfate–polyacrylamide gel electrophoresis (SDS-PAGE)and transblotted onto PVDF membranes. The membranes were then blocked with 5% milk and incubated at 4°C overnight with the following primary antibodies: anti- STAT3 (Cell Signaling Technology, Danvers, MA; Cat. #9139), anti-phosphorated-STAT3 (Cell signaling; Cat. #9145), or anti-β-actin antibody (Sigma-Aldrich; Cat. #A5316). The bands were visualized by enhanced chemiluminescence (Thermo Fisher Scientific) and quantified by densitometry analysis.

### ELISA

Ileal levels of IL-22 were determined by R&D systems ELISA kit (Minneapolis, MN; Cat. #M2200) following the manufacturer’s recommendation.

### Immunofluorescence

The levels of IL-22 in mouse ileum after alcohol exposure, hepatic inflammation status with or without IL-22 treatment, and gut barrier function in mouse ileum and isolated organoids were examined by immunofluorescence staining. Cryostat sections of mouse ileum or liver tissues were incubated with anti-IL-22 (Genetex, Irvine, CA; Cat. #GTX18498), anti-F4/80 (BD Biosciences, Franklin Lakes, NJ; Cat. #565409), anti-MPO (Lifespan Biosciences, Seattle, WA; Cat. #LS-B6699), anti-DEFA5 (Elabscience, Houston, TX; ESAP13305), anti-ZO-1 (Millipore, Burlington, MA; Cat. #MABT11), anti-Ki67 antibody (Millipore; Cat. #AB9260), or anti-sodium hydrogen antiporter 3 (NHE3) antibody (Novus Biologicals, Centennial, CO; Cat. # NBP1-82574) followed by Alexa Fluor 594-conjugated IgG antibodies (Jackson ImmunoResearch Laboratories, West Grove, PA).

### Statistics

All data are expressed as mean ± standard deviation (SD). Statistical analysis was carried out using two-tailed Student’s *t*-test or one-way ANOVA followed by a *post hoc* Tukey test where appropriate using GraphPad Prism software (La Jolla, CA). ^*^ *P* < 0.05, ^**^ *P* < 0.01, ^***^ *P* < 0.001.

## Results

### Alcohol exposure reduces intestinal IL-22 and recombinant IL-22 treatment alleviates alcohol-induced liver damage

**Figure 1:**
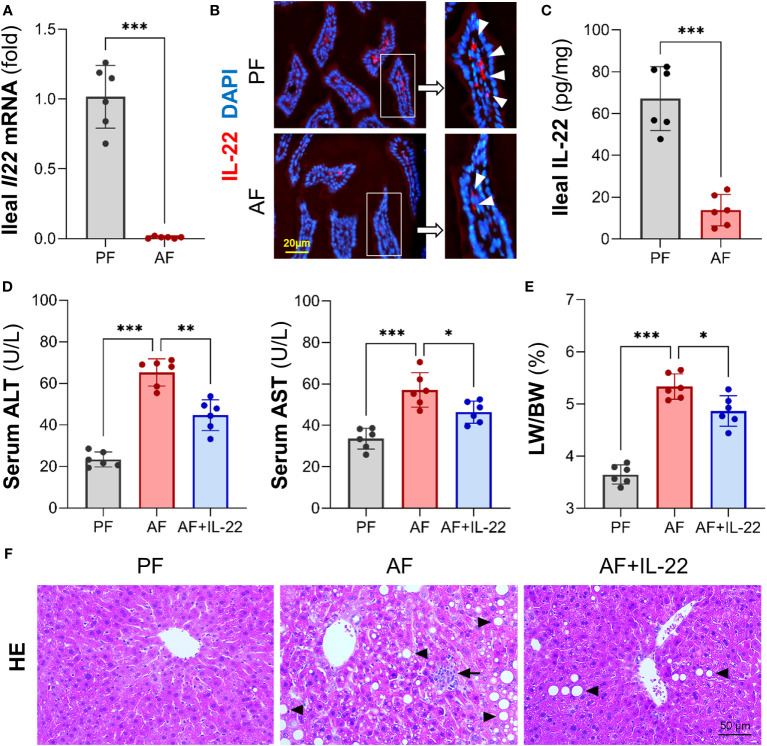
Chronic alcohol feeding reduces intestinal IL-22 and administration of IL-22 alleviates alcoholic hepatitis in mice. C57BL/6J WT mice were fed Lieber-DeCarli liquid diets containing alcohol (alcohol-fed, AF) or isocaloric dextran (pair-fed, PF) for 8 wk. **(A)** The mRNA levels of ileal IL-22. **(B)** Representative immunofluorescence (IF) staining of ileal IL-22 (red). Nuclei were counterstained by DAPI (blue). Scale bar, 20 µm. **(C)** Ileal IL-22 quantified by ELISA. Recombinant mouse IL-22 was treated to AF mice through intraperitoneal (i.p.) injection at 1 mg/kg every other day for the last 2 wk in an 8-wk alcohol feeding experiment. **(D)** Serum ALT and AST levels. **(E)** Liver weight to body weight ratio. **(F)** Hematoxylin and eosin (H&E) staining of mouse liver sections. Arrowheads indicate lipid accumulation and arrows indicate inflammatory cells. Scale bar, 50 μm. ^*^ *P* < 0.05, ^**^ *P* < 0.01, ^***^ *P* < 0.001.

Our previous study demonstrated that Paneth cell-produced α-defensins were significantly reduced upon alcohol exposure in a mouse model of AH (6). To explore the cause of alcohol-induced AMP reduction, we first determined intestinal IL-22 levels and found that alcohol exposure significantly reduced ileal mRNA encoding for IL-22 in mice (**Figure 1A**). Compared to PF mice, AF mice had a significant reduction in IL-22^+^ staining in the ileal lamina propria (**Figure 1B**). The protein levels of IL-22 averaged 67.18 ± 15.32 pg/mg in PF mice and was decreased to 13.84 ± 7.45 pg/mg in AF mice (**Figure 1C**).

To determine whether IL-22 may reverse alcohol-induced organ damage at the gut-liver axis, we then treated AF mice with recombinant mouse IL-22 every 2 days starting from day 1 of the 7^th^ wk of alcohol feeding till the end of the feeding. Treatment with IL-22 significantly suppressed alcohol-induced serum levels of alanine aminotransferase (ALT) and aspartate aminotransferase (AST) levels (**Figure 1D**). IL-22 also slightly decreased the ratio of liver to body weight that was significantly elevated by alcohol (**Figure 1E**). Compared to PF group, AF group showed substantial lipid droplets accumulation (**Figure 1F**; arrowheads) and inflammatory cell infiltration (**Figure 1F**; arrows) in the liver, which were both improved by IL-22 intervention.

**Figure 2:**
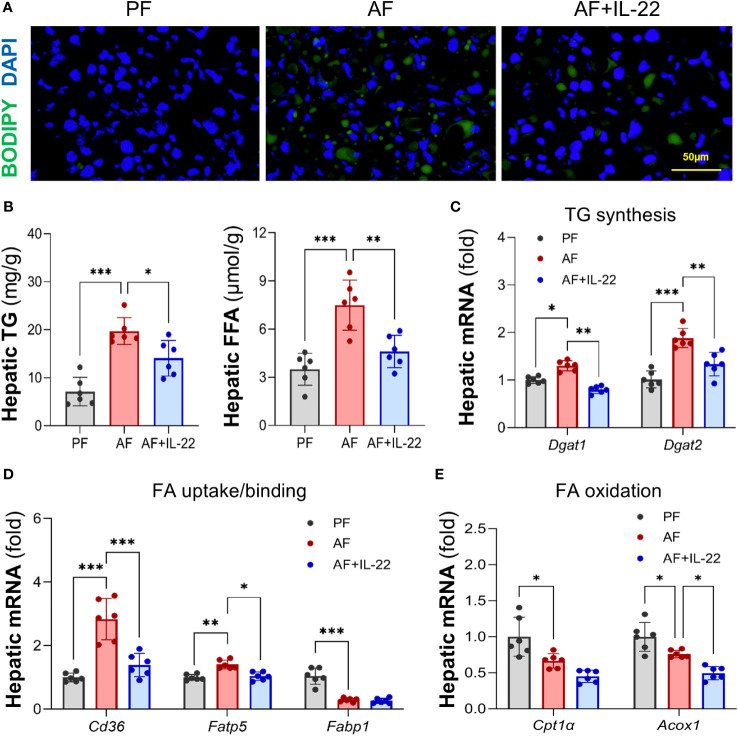
Administration of IL-22 reduces alcohol-induced lipid accumulation in mouse liver. **(A)** BODIPY 493/503 staining of mouse liver. Neutral lipids were stained in green, and nuclei were counterstained in blue. Scale bar, 50 μm. **(B)** Hepatic TG and FFA levels. **(C–E)** Expressions of hepatic proteins involved in lipid metabolism, including **(C)** TG synthesis, **(D)** FA uptake and binding and **(E)** FA oxidation. ^*^ *P* < 0.05, ^**^ *P* < 0.01, ^***^ *P* < 0.001. PF, pair-fed; AF, alcohol-fed.

BODIPY 493/503 staining further confirmed that IL-22 treatment effectively reversed alcohol-induced hepatic accumulation of neutral lipid droplets (**Figure 2A**). Quantification of lipids showed that alcohol consumption resulted in excessive TG and FFA depositions in mouse liver, whereas IL-22 treatment reduced hepatic TG and FFA levels by 28.7% and 38.5%, respectively, compared with AF group (**Figure 2B**). Alcohol-induced expression of TG synthesis enzymes, *Dgat1* (diglyceride acyltransferase 1) and *Dgat2*, were reversed by IL-22 treatment (**Figure 2C**). Alcohol upregulated the expressions of FA uptake transporters, *Cd36* and *Fatp5* (fatty acid transport protein 5), which were reduced/normalized by IL-22 (**Figure 2D**). IL-22 treatment did not further affect alcohol-downregulated FA binding protein *Fabp1* (fatty acid binding protein 1; **Figure 2D**) nor FA oxidation enzymes *Cpt1α* (carnitine palmitoyltransferase 1α; **Figure 2E**). There was a light reduction in mRNAs encoding Acox1 in AF+IL-22 group compared to that in AF group (**Figure 2E**). Proteins involved in very-low-density lipoprotein (VLDL) secretion, such as *Apob* (apolipoprotein B) and *Mtp* (Microsomal triglyceride transfer protein), showed no difference at mRNA levels among the groups (data not shown).

**Figure 3:**
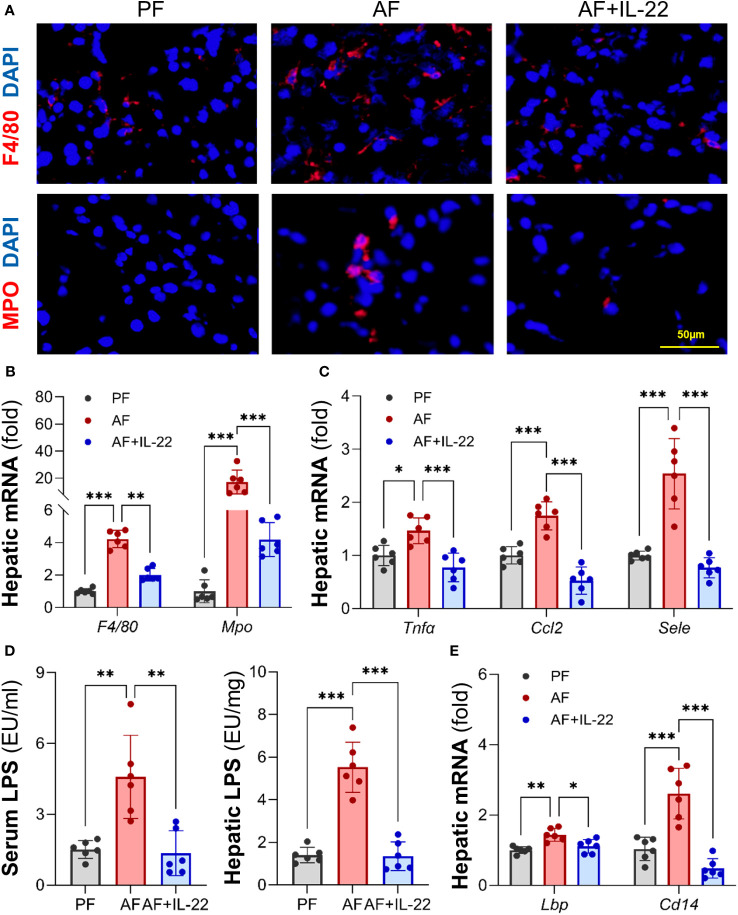
Administration of IL-22 reduces alcohol-induced hepatic inflammatory response and LPS translocation. **(A)** Representative IF staining of F4/80^+^ macrophages and MPO^+^ neutrophils in the liver. Scale bar, 50 μm. **(B)** Expressions of hepatic *F4/80* and *Mpo*. **(C)** Expressions of hepatic pro-inflammatory cytokines and chemokines. **(D)** Serum and hepatic LPS levels. **(E)** The mRNA levels of LPS signaling molecules in the livers of mice. ^*^ *P* < 0.05, ^**^ *P* < 0.01, ^***^ *P* < 0.001. PF, pair-fed; AF, alcohol-fed.

The effects of IL-22 on countering alcohol-induced liver inflammation were further analyzed. Unlike AF mice, which had dramatically increased positive staining of inflammatory cells, such as F4/80^+^ macrophages and MPO^+^ neutrophils, in the liver compared with PF mice, AF mice with IL-22 treatment exhibited significantly fewer hepatic inflammatory cells (**Figure 3A**; Upper: F4/80^+^, lower: MPO^+^). The mRNA levels of *F4/80* and *Mpo* correlated with the immunofluorescence findings that both were significantly upregulated by alcohol and reversed by IL-22 (**Figure 3B**). In line with that, IL-22 treatment decreased hepatic mRNA levels of pro-inflammatory cytokines and chemokines, including *Tnfα*, *Ccl2*, and *Sele*, compared with AF only group (**Figure 3C**). To explore whether the observed anti-inflammatory effect of IL-22 is associated with bacterial-derived signaling, we measured blood and hepatic levels of LPS, a representative marker of translocated PAMPs. As shown in **Figure 3D**, IL-22 normalized blood and hepatic LPS levels that were significantly elevated by alcohol. Examination of LPS signaling molecules in the liver further revealed that alcohol stimulated the expression of *Lbp* (LPS binding protein) and *Cd14* (cluster of differentiation 14), which were both inhibited by IL-22 treatment (**Figure 3E**).

### Recombinant IL-22 orchestrates gut microbiota favoring the enrichment of Akkermansia to combat alcohol-induced gut dysbiosis

**Figure 4:**
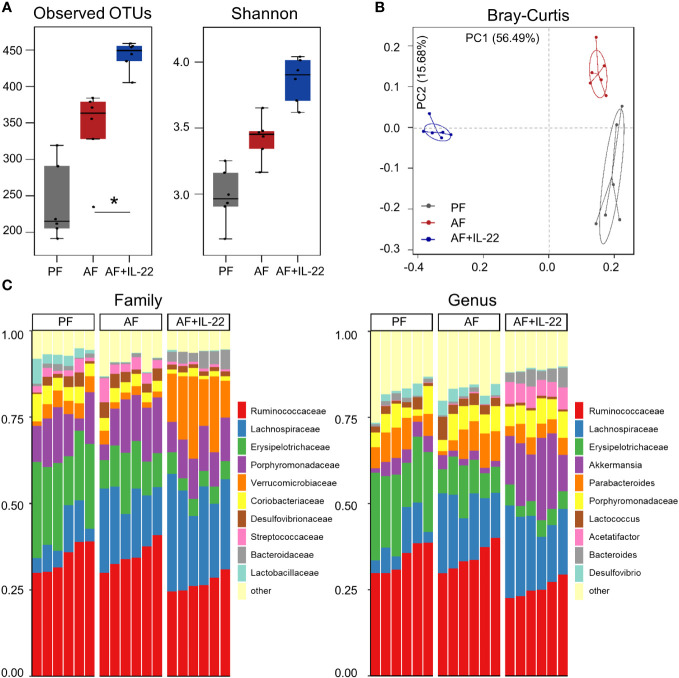
IL-22 treatment increases mouse cecal bacteria diversity and shifts the composition that are perturbed by alcohol. **(A)** Alpha-diversity measurements of observed number of OTUs and Shannon index. ^*^ *P* < 0.05. **(B)** PCoA plot showing dissimilarity in bacterial community structures based on Bray-Curtis distances. **(C)** Barplot showing the cecal bacterial compositions at family level (left) and genus (right) level, respectively. PF, pair-fed; AF, alcohol-fed.

The observed hepatic protective effects of IL-22 on restraining PAMP translocation motivated us to explore how IL-22 impacts the gut microbiota in terms of alcohol intoxication. Cecal microbiota was analyzed by metagenomic sequencing of the bacterial 16S rRNA gene. Alpha-diversity analysis showed a trend of elevation in microbial richness (*P* = 0.253 for observed OTUs index and *P* = 0.175 for Shannon) in AF mice compared with PF mice, while IL-22 treatment to AF mice had the highest levels of both alpha-diversity indexes (**Figure 4A**). Both alcohol and IL-22 treatment altered the structure of microbial communities as determined by β-diversity analysis. As shown in **Figure 4B**, Bray-Curtis distance matrices clearly separated all 3 groups into different clusters (ANOSIM test, *P* < 0.001, r = 0.930). We then compared the bacterial abundance between groups at the family and genus levels (**Figure 4C**). At the family level, Verrucomicrobiaceae is the family that showed the largest changes when comparing AF+IL-22 group with AF group. Its abundance was 3.87 ± 1.28% in PF mice, reduced to 2.25 ± 0.96% in AF mice, and dramatically increased to 17.00 ± 5.20% in AF+IL-22 mice (**Figure 4C** left, colored in orange). Meanwhile, IL-22 significantly enriched alcohol-inhibited Bacteroidaceae and reduced alcohol-increased Streptococcaceae. At the genus level, a similar trend was observed that *Akkermansia*, which belongs to Verrucomicrobiaceae, was largely expanded in the gut after IL-22 treatment (**Figure 4C** right, colored in purple).

**Figure 5:**
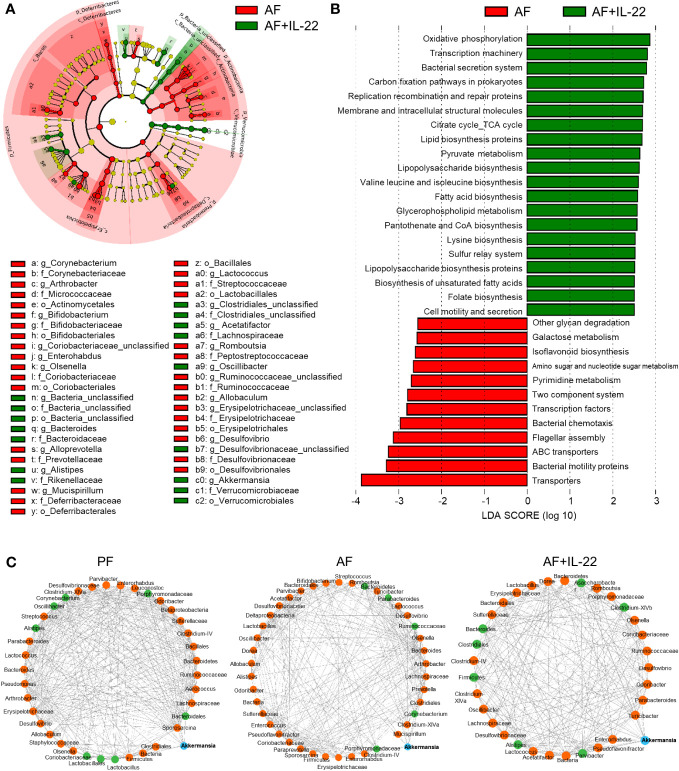
Administration of IL-22 results in distinct cecal microbial and functional variations other than that associated with alcohol intoxication in mice. **(A)** Cladogram taxonomic abundances of bacteria. Legend of prominent taxons is shown at bottom. **(B)** Bacterial gene functions predicted based on 16S rRNA gene sequences using the PICRUSt algorithm and annotated from KEGG databases. Red: bacterial genera enriched in AF mice; Green, bacterial genera enriched in AF+IL-22 mice. **(C)** Bacterial co-occurrence network revealed the key role of *Akkermansia* spp. in formation of bacterial correlation network in intestinal microbiota. *Akkermansia* spp. is highlighted as blue circle, while those genera directly correlated with *Akkermansia* spp. are colored as green. Solid line, positive correlation; dash line, negative correlation.

The phylotypes in gut microbiota between AF and AF+IL-22 mice were further evaluated by LEfSe analysis. The cladogram showed a compelling effect of IL-22 on inhibiting a panel of bacteria in phyla of Firmicutes, Actinobacteria, and Proteobacteria, such as Deferribacteraceae, Erysipelotrichaceae, Coriobacteriaceae, Bifidobacteriaceae, Micrococcaceae, Corynebacteriaceae, and Desulfovibrionaceae when treated to AF mice (**Figure 5A**; Red, IL-22-decreased; Green, IL-22-increased). AF+IL-22 mice were also characterized by preponderant Verrucomicrobia and Bacteroidetes abundance. Metagenomic analysis of functional bacterial genes defined 32 dominant Kyoto Encyclopedia of Genes and Genomes (KEGG) pathways that were markedly different between AF and AF+IL-22 groups, among which 12 KEGG pathways were more abundant in AF group and 20 KEGG pathways were more abundant in AF+IL-22 group (**Figure 5B**). Genes mapping for microbial energy metabolism, including oxidative phosphorylation, citric acid cycle, and pyruvate metabolism, were more frequent in AF+IL-22 mice compared with AF mice. On the other hand, bacterial genes involved in triggering host inflammatory responses, such as bacterial motility proteins, flagellar assembly, bacterial chemotaxis, and two component system, were fewer in AF+IL-22 group than in AF group. Next, we analyzed the corresponding co-occurrence bacterial networks and found that PF group had a network density of 0.516, whereas the inter-genera correlation in AF group was much lower with a network density of 0.358. IL-22 treatment partially restored the network density to 0.453. Furthermore, the number of genera participating in the core interaction network was increased from 38 in PF group to 42 in AF group, while IL-22 treatment reduced the number to 33. With the consistent observations in a reduction of *Akkermansia* in patients and animal models of ALD (6, 37) as well as its compelling enrichment induced by IL-22 treatment (**Figure 4C**), we further analyzed sub-networks related to *Akkermansia*. The numbers of genera that were directly correlated with *Akkermansia* were 8, 5, and 7 for PF, AF, and AF+IL-22 groups, respectively (**Figure 5C**; Blue, *Akkermansia*; Green, bacteria directly correlated with *Akkermansia*), suggesting that IL-22 drives gut microbiota toward a beneficial direction in the host favoring the improvement of alcoholic hepatitis.

### Recombinant IL-22 treatment improves alcohol-impaired AMP production, gut barrier, and intestinal STAT3 signaling

**Figure 6:**
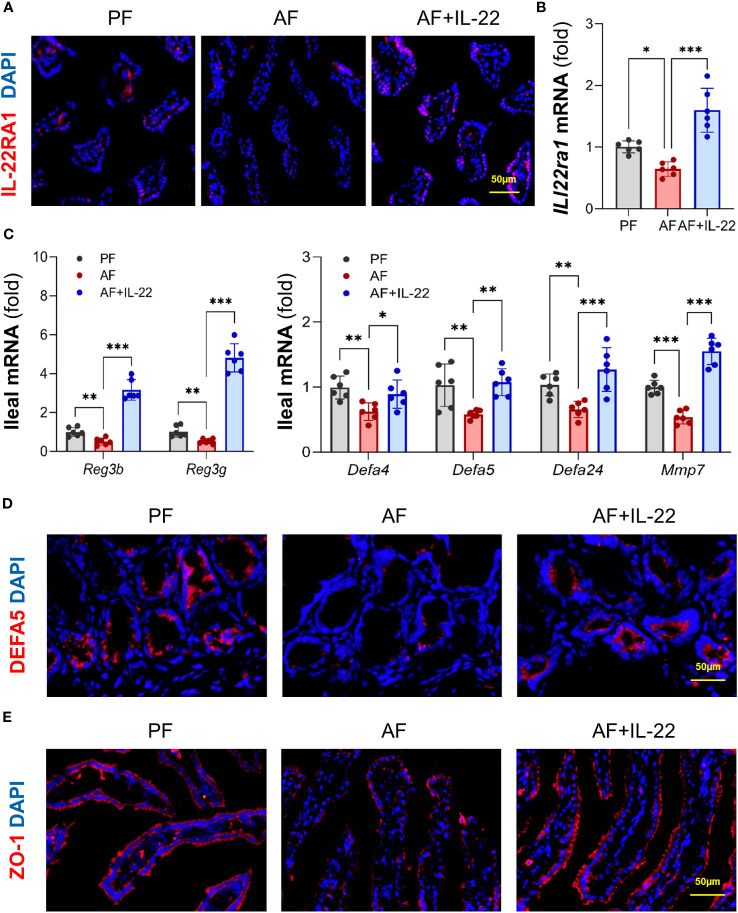
IL-22 treatment reverses alcohol-reduced epithelial AMP production and improves alcohol-disrupted gut barrier. **(A)** IF staining of ileal IL-22RA1. Positive signals of IL-22RA1 were stained in red, and nuclei were stained by DAPI in blue. Scale bar, 50 μm. **(B)** The mRNA levels of ileal *Il22ra1*. **(C)** The mRNA levels of ileal AMPs. **(D, E)** IF staining of ileal DEFA5 **(D)** and ZO-1 **(E)**. DEFA5 or ZO-1 was stained in red, while nuclei were stained by DAPI in blue. Scale bar, 50 μm. ^*^ *P* < 0.05, ^**^ *P* < 0.01, ^***^ *P* < 0.001. PF, pair-fed; AF, alcohol-fed.

We then analyzed intestinal IL-22RA1 to validate if recombinant IL-22 could directly activate its receptor and elicit intestinal protection. Compared to PF group, AF group showed reduced intestinal IL-22RA1^+^ immunofluorescence staining intensity, whereas IL-22 treatment restored the signal strength (**Figure 6A**). Similarly, the expression of Il22ra1 was decreased after alcohol exposure and upregulated by IL-22 treatment (**Figure 6B**), indicating that recombinant IL-22 reached to the intestine and activated its receptor to induce local responses.

Given the fact that host AMPs critically regulate gut microbiota, we next determined whether the ability of IL-22 in re-establishing gut microbiome was related to AMP regulation. Consistent with a previous report (18), intestinal Reg3β and Reg3γ were both inhibited by alcohol and significantly increased by IL-22 treatment (**Figure 6C**). Moreover, since our group recently reported that another important type of AMPs, Paneth cell α-defensins, were also decreased by alcohol (6), we determined the changes of α-defensins and found that IL-22 treatment also effectively reversed alcohol-downregulated expression of α-defensins, including *Defa4*, *Defa5*, and *Defa24*, as well as their activation factor, *Mmp7* (matrix metalloproteinase 7; **Figure 6C**). Subsequent immunofluorescence staining of DEFA5 revealed that alcohol-reduced distribution of DEFA5 in the small intestinal crypts were restored by IL-22 treatment (**Figure 6D**). Gut barrier integrity was determined by examining tight junction protein, ZO-1. Compared to PF mice which displayed a continuous circumferential distribution of ZO-1 along the apical part of the intestinal epithelium, reduced fluorescent intensity and disassembly of ZO-1 were found in AF mice, which was markedly improved in AF+IL-22 mice (**Figure 6E**). Thus, IL-22 treatment reversed alcohol-induced intestinal damage, at least partially via regulating AMP production and epithelial barrier integrity.

### IL-22 directly regulates intestinal AMP production through STAT3 signaling

**Figure 7:**
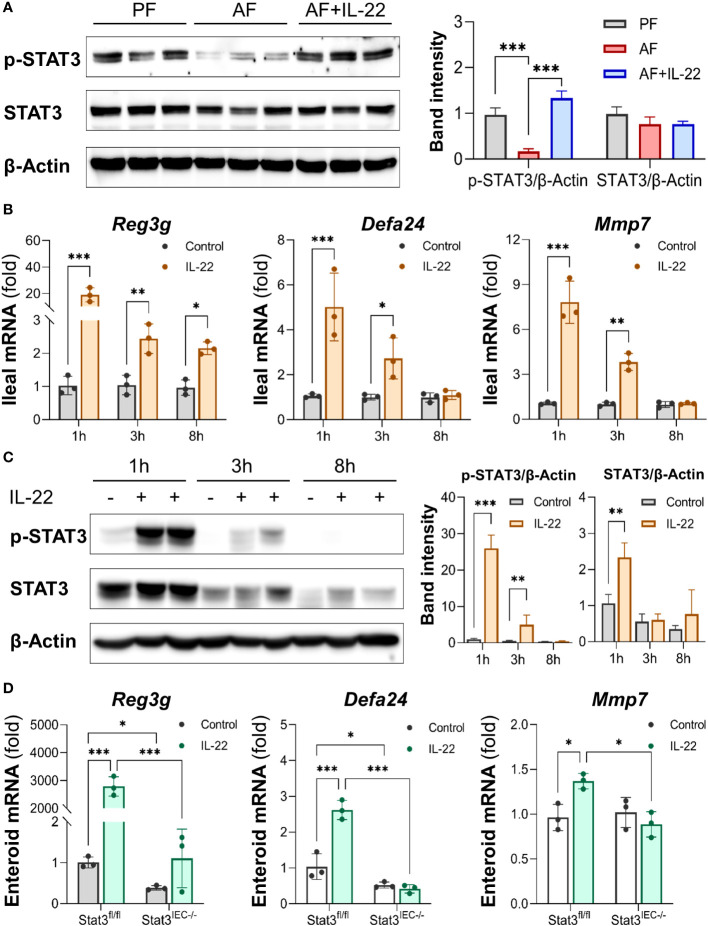
IL-22 directly induces intestinal AMP expression via activating STAT3 regardless of microbiota. Recombinant mouse IL-22 was treated to AF mice through intraperitoneal (i.p.) injection at 1 mg/kg every other day for the last 2 wk in an 8-wk alcohol feeding experiment. **(A)** WB and quantification of mouse ileal phospho-STAT3 and STAT3. WT mice were given either recombinant mouse IL-22 at 1 mg/kg or same volume of saline for 1, 3, or 8 h. **(B)** The mRNA levels of ileal AMPs and MMP7. **(C)** WB and quantification of mouse ileal phospho-STAT3 and STAT3. Small intestinal organoids isolated from Stat3^fl/fl^ and Stat3^IEC-/-^ mice were treated with 100 ng/ml IL-22 for 10 h. **(D)** The mRNA levels of AMPs and MMP7 in organoids isolated from Stat3^fl/fl^ and Stat3^IEC-/-^ mice after IL-22 treatment. ^*^ *P* < 0.05, ^**^ *P* < 0.01, ^***^ *P* < 0.001.

Intestinal STAT3 signaling was examined to explore its role in IL-22-induced intestinal protection in ALD. Alcohol suppressed STAT3 phosphorylation in the small intestine without affecting total STAT3 protein levels as indicated by Western blot, whereas recombinant IL-22 reactivated intestinal STAT3 (**Figure 7A**). To characterize the regulation of IL-22 on intestinal AMPs and STAT3 signaling, we performed an acute study in which single dose of recombinant IL-22 was given to WT mice, and AMPs and STAT3 were measured at different time points post treatment. As illustrated in **Figure 7B**, acute IL-22 treatment prompted fast and robust gene expression related to intestinal AMPs, including *Reg3g*, *Defa24* and *Mmp7*, as early as 1 h post IL-22 treatment and lasted for up to 8 h. The expressions of AMPs were highest at 1 h compared to 3 h and 8 h. Meanwhile, intestinal STAT3 was significantly phosphorylated and activated by IL-22 at 1 h post treatment and returned to baseline by 8 h (**Figure 7C**).

Next, we generated IEC-specific STAT3 knockout mice and established corresponding small intestinal organoids to further explore the involvement of STAT3 in IL-22-mediated AMP expression and to exclude the involvement of gut microbiota. Treatment with IL-22 to small intestinal organoids strikingly stimulated the expression of *Reg3g*, *Defa24*, and *Mmp7* in WT organoids. In Stat3^IEC-/-^ organoids, the basal levels of *Reg3g* and *Defa24* were lower than those in WT organoids, and IL-22-induced expression of *Reg3g*, *Defa24* and *Mmp7* were all inhibited (**Figure 7D**). Therefore, we concluded that IL-22 regulates intestinal AMP expression through STAT3 signaling, and the induction is independent of gut microbiota.

### IL-22 stimulates intestinal epithelium differentiation that is impaired by alcohol

**Figure 8:**
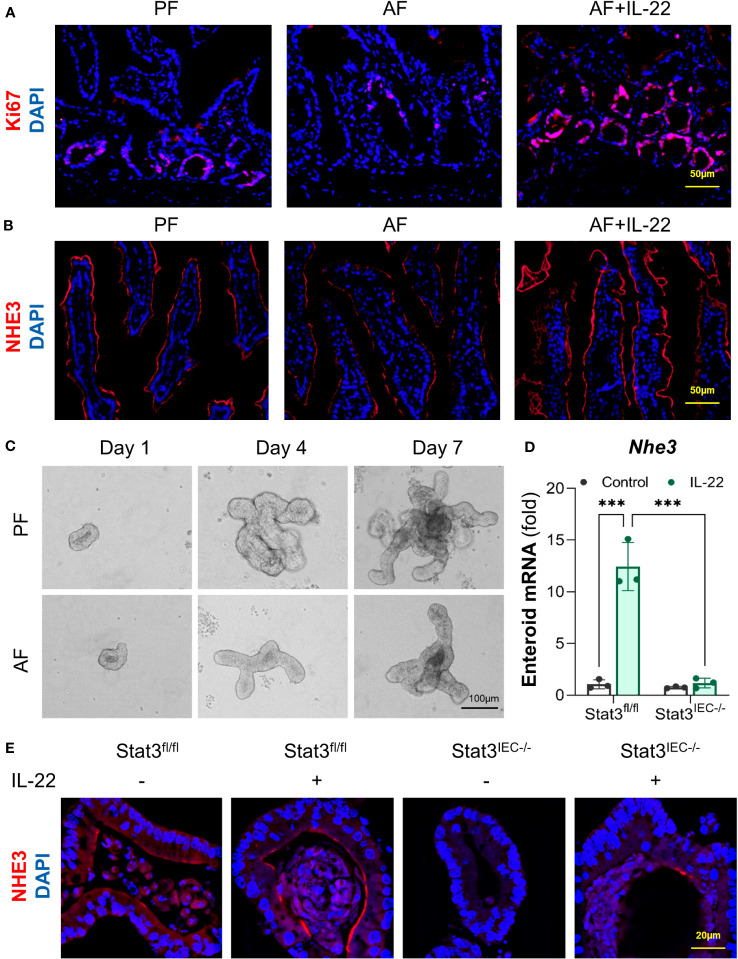
IL-22 stimulates IEC differentiation in a STAT3-depedent manner in mice and cultured organoids. WT mice were subjected to an 8-wk alcohol feeding, and IL-22 was i.p. injected at 1 mg/kg for the last 2 wk of feeding. **(A)** IF staining of ileal Ki67 (red) and nuclei (blue). Scale bar, 50 μm. **(B)** IF staining of ileal NHE3 (red) and nuclei (blue). Scale bar, 50 μm. **(C)** Organoid forming efficiency assessed by bright field imaging. Scale bar, 100 μm. Small intestinal organoids isolated from Stat3^fl/fl^ and Stat3^IEC-/-^ mice were treated with 100 ng/ml IL-22 for 10 h. **(D)** The mRNA levels of *Nhe3* in organoids isolated from Stat3^fl/fl^ and Stat3^IEC-/-^ mice after IL-22 treatment. ^***^ *P* < 0.001. **(E)** IF staining of NHE3 (red) in organoids after IL-22 treatment. Nuclei were stained by DAPI (blue). Scale bar, 20 μm.

Lastly, we investigated the effects of IL-22 on intestinal epithelial cell differentiation in mice exposed to alcohol and in cultured organoids with or without STAT3. In the crypts of small intestine of AF mice, the number of Ki67^+^ cell was lower than that in PF mice, whereas that was dramatically raised in AF+IL-22 mice, suggesting an upregulated epithelial proliferation induced by IL-22 (**Figure 8A**). NHE3 is widely used as a functional marker of differentiated IECs (38), and examination of ileal NHE3 revealed reduced fluorescent intensity of NHE3^+^ staining at the apical part of IECs in AF mice compared with PF mice, whereas the strongest NHE3^+^ staining were observed in AF+IL-22 mice (**Figure 8B**). Small intestinal organoids isolated from PF and AF mice showed an inhibitory effect of alcohol on organoid budding time in culture (**Figure 8C**). IL-22 treatment to cultured organoids directly stimulated the expression of *Nhe3* in WT organoids up to 12-fold, which was completely blocked by IEC-specific STAT3 deletion (**Figure 8D**). Furthermore, immunofluorescence staining of NHE3 confirmed that IL-22-induced epithelial NHE3 expression is STAT3-dependent (**Figure 8E**). Immunofluorescence staining of NHE3 showed that IL-22 stimulated NHE3 expression in WT organoids. Compared to the levels of NHE3^+^ staining in WT organoids, NHE3^+^ staining intensities were much lower in Stat3^IEC-/-^ organoids with or without IL-22 treatment (**Figure 8E**). Taken together, besides its role in the regulation of intestinal AMPs, IL-22 also promotes intestinal epithelium differentiation that is inhibited by alcohol intoxication, which represents another line of protection in the intestine to prevent systemic PAMP translocation induced by alcohol.

## Discussion

**Figure 9:**
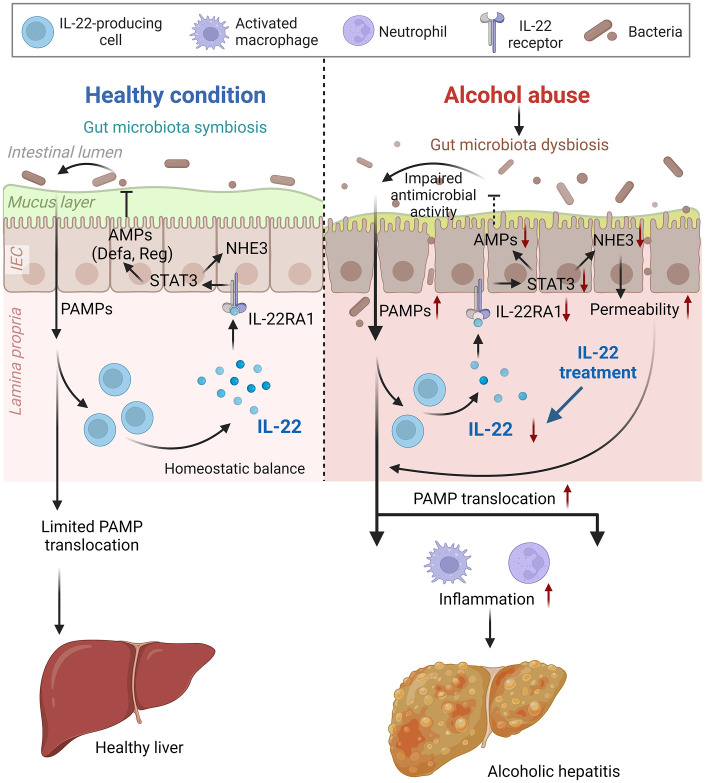
Schematic figure summarizing the major findings of the present study (created with BioRender.com). AMP, antimicrobial peptide; IEC, intestinal epithelial cells; NHE3, sodium-hydrogen exchanger 3; PAMP, pathogen-associated molecular pattern; STAT3, signal transducer and activator of transcription 3.

Altered gut microbial ecosystem is associated with metabolic and immune disorders, including alcoholic hepatitis, obesity, and metabolic-dysfunction-associated steatotic liver disease (39). Upon alcohol intoxication, the interplay between the host and gut microbiota is perturbed by mechanisms that remain incompletely understood. IL-22 is increasingly recognized in promoting intestinal homeostasis (14, 16, 17), and previous studies reported that short-term alcohol exposure reduces intestinal IL-22 levels in rodents (18, 40). The study demonstrates the protective effects of IL-22 in both the intestine and the liver against alcohol toxicity in an 8-week alcohol feeding mouse model (summarized in **Figure 9**). We found that intestinal IL-22-STAT3 signaling is an important regulator for intestinal AMP production, gut microbiota symbiosis, and epithelial barrier function that are disrupted by alcohol. The study provided experimental evidence that IL-22 treatment is effective in alleviating alcohol-induced PAMP translocation and hepatic inflammation.

IL-22 has been proposed as a promising therapeutic target in treating many gastrointestinal diseases (41). The direct hepatoprotective effects of IL-22 have been investigated in multiple models of liver injuries, including T cell-mediated hepatitis (42), acetaminophen-induced liver injury (43, 44), fatty liver disease (45–47), and AH (48). A previous study revealed that IL-22-mediated hepatoprotection in ALD is involved in the stimulation of proliferation, inhibition of apoptosis, and prevention of fatty acid uptake in hepatocytes (48). Our current study indicated that IL-22 downregulates hepatic expression of several lipid metabolism genes involved in TG synthesis and fatty acid uptake, which may involve both direct effect of IL-22 to the liver and indirect signals from the gut-derived microbial products, such as LPS. In fact, LPS has been reported to directly and/or synergistically induce lipid disorder and promote fatty liver (49, 50). An earlier study found that suppression of LPS binding protein improves obesity-associated fatty liver (51).

Our study revealed a profound protective role of IL-22 in the intestine following alcohol exposure via regulating AMP production (both Reg3 and α-defensins), promoting the growth of commensal bacteria, and enhancing gut barrier function. Hendrikx et al. reported an intestinal protective effect of IL-22 against AH using bioengineered bacteria overexpressing IL-22 (18). The authors found that treatment with IL-22-producing *L. reuteri* upregulated intestinal Reg3γ levels and alleviated alcohol-induced liver injury. Administration of IL-22 was also protective in a mouse model of ethanol and burn injury (40). Although the whole story for IL-22-mediated beneficial effects on the intestine is not completely understood, our findings provide more comprehensive knowledge supporting the feasibility of IL-22 in clinical use for AH treatment.

One of the important findings in the study is that IL-22 directly regulates the expression and activation of α-defensins. Previous studies investigating the effects of IL-22 on AMP induction mostly focused on a few preselected AMPs, such as Reg3 (18). In the present study, in addition to the Reg3 family, we also measured α-defensins and their activation factor, MMP7, in mouse models of chronic alcohol feeding with IL-22 treatment, in acute IL-22 administration, and in small intestinal organoids. We observed that IL-22-mediated α-defensin expression is independent of gut microbiota. Unlike most AMPs that require bacterial stimulation for robust production, α-defensins are constitutively expressed even in germ-free mice (52), which suggests a fundamental role of α-defensins in intestinal homeostasis. However, the regulatory mechanisms of α-defensin expression are still unclear. By implementing the IEC-specific STAT3 knockout mouse model, we identified that IL-22 induces α-defensin expression through STAT3.

IL-22 impacts not only pathogenic bacteria to prevent infection, but also commensal gut microbiota to orchestrate microbial symbiosis (22, 23). In IL-22RA1 deficient mice, a dramatic depletion of *Lactobacillaceae*, *Bifidobacteriaceae*, and *Ruminococcaceae* families and an overgrowth of opportunistic pathogen *Enterococcus* spp. were detected (22). To the best of our knowledge, this study is the first report showing that IL-22 induces robust bloom of commensal *Akkermansia* spp. in the gut. *Akkermansia* spp. is commonly found in the gut of human and mouse, ranging 2-5% of the microbial community (53). We found that the bacterium makes up to 17% of the total cecal microbiota after IL-22 administration. *Akkermansia* spp. is more abundant in healthy subjects than in patients with obesity and diabetes (54), inflammatory bowel diseases (55), and ALD (37). Accumulating evidence from animal and human studies suggest that *Akkermansia* has a promising potential for the prevention and treatment of diseases, including ALD (37). The precise mechanism by which IL-22 exerts *Akkermansia* spp. expansion is obscure. One proposed mechanism could be a better nourished microenvironment provided by IL-22 that promotes the growth of *Akkermansia*. IL-22 is known to regulate many mucus-associated genes, such as mucin-1 (MUC1), MUC3, MUC10, and MUC13 (16), whereas *Akkermansia* spp. is a mucin-degrading bacterium (56). Another possibility is the regulatory effect of AMPs, especially α-defensins. Besides direct bacterial killing, AMPs have been recently recognized to possess immunoregulatory effects, such as neutralizing pathogenic toxins, modulating immune cell differentiation, and initiating adaptive immunity (10). We previously reported that synthetic HD5 treatment to AF mice induced *Akkermansia* spp. in mouse cecum for over 10 fold (6), a phenomenon similar to what was observed in IL-22-treated AF mice. Interestingly, another group reported that HD5 treatment to non-treated WT mice also dramatically increased *Akkermensia* spp. without altering microbial diversity (57). A third mechanism would be a secondary effect of IL-22-improved gut microenvironment that is dampened by alcohol. However, this hypothesis cannot explain why the levels of *Akkermansia* spp. in alcohol plus IL-22 group were further elevated. Therefore, future studies are needed to explore the causal-effect mechanism of how IL-22 promotes *Akkermansia* spp. expansion. Our findings that IL-22 treatment favors *Akkermansia* spp. provide new insights into IL-22-mediated protection against AH at the gut-liver axis.

Although it has been reported that IL-22 regulates gut barrier function, the precise functional mechanisms of IL-22 in the intestine remain largely unknown. Here, we report that NHE3 is a direct target of IL-22 via STAT3 signaling, which is impaired by alcohol exposure. NHE3 is used as a marker for differentiated epithelium (38). It plays an essential role in maintaining sodium and water homeostasis in the intestine yet the regulatory mechanisms of NHE3 are not well-defined (58). It is worth mentioning that NHE3 is essential for gut microbial symbiosis as mice deficient in NHE3 have microbial dysbiosis and develop spontaneous colitis (59, 60). Hence, we believe that IL-22-mediated NHE3 induction not only indicates a well-differentiated gut barrier that prevents the penetration of PAMPs but also provides another mechanism that orchestrates gut microbiota. The crosstalk between IL-22, epithelial NHE3, gut microbiota, and even AMPs in the pathogenesis of AH warrants further investigation.

It remains largely unknown how alcohol reduces intestinal IL-22 levels. IL-22 is mainly produced by T helper type 17 (Th17), Th22, and innate lymphoid type 3 (ILC3) cells. Other potential IL-22-producing cells include γδ T cells, natural killer cells, and neutrophils (14–16). IL-22-expressing ILC3 cells were reported to be significantly reduced without affecting the levels of IL-22 repressor, IL-25, in mice after alcohol exposure (18). In the same study, intestinal levels of indole-3-acetic acid (IAA), which is an aryl hydrocarbon receptor (AhR) ligand that regulates IL-22 expression, was reduced by alcohol feeding (18). More work is warranted to explore the cellular and molecular mechanisms of intestinal IL-22 reduction in the pathogenesis of ALD. In conclusion, our data indicate that administration of IL-22 promotes intestinal innate defense via regulating AMP production, orchestrating gut microbiota symbiosis, and stimulating IEC differentiation, all of which contribute to a profound effect on inhibiting the translocation of gut-derived PAMPs and subsequent hepatic inflammation. The study strongly supports the potential clinical utility of IL-22 targeting both the intestine and liver in ALD treatment. Persistently upregulated IL-22 can promote inflammation and accelerate tumor growth (61, 62). Elucidating factors determining whether IL-22 is protective or inflammatory is of high priority and necessitates further investigation.

## Data availability statement

The original contributions presented in the study are publicly available. This data can be found here: National Center for Biotechnology Information Short Read Archive Database with BioProject accession number PRJNA1022827.

## Ethics statement

The animal study was approved by North Carlina Research Campus Institutional Animal Care and Use Committee. The study was conducted in accordance with the local legislation and institutional requirements.

## Author contributions

RY: Data curation, Formal Analysis, Investigation, Methodology, Writing – original draft, Writing – review & editing. XW: Data curation, Formal Analysis, Investigation, Software, Writing – review & editing. LH: Data curation, Investigation, Writing – review & editing. HD: Data curation, Investigation, Writing – review & editing. WG: Data curation, Investigation, Writing – review & editing. XS: Data curation, Writing – review & editing. JZ: Data curation, Resources, Writing – review & editing. ZZ: Funding acquisition, Resources, Supervision, Writing – review & editing. WZ: Conceptualization, Formal Analysis, Funding acquisition, Investigation, Resources, Supervision, Writing – review & editing.
